# Identification of the *PmNAC* Gene Family in *Pinus massoniana*: *PmNAC82* Modulates Wood Biosynthesis by Activating SCW-Related Genes

**DOI:** 10.3390/plants15101568

**Published:** 2026-05-21

**Authors:** Sheng Yao, Yidan Song, Qianzi Li, Yu Chen, Xiang Cheng, Dengbao Wang, Qiong Yu, Kongshu Ji

**Affiliations:** 1 State Key Laboratory of Tree Genetics and Breeding, Nanjing Forestry University, Nanjing 210037, China; yaosheng0817@163.com (S.Y.); syd@njfu.edu.cn (Y.S.); qianzili@njfu.edu.cn (Q.L.); chenyu980905@163.com (Y.C.); chengxiang@njfu.edu.cn (X.C.); dbw@njfu.edu.cn (D.W.); 2 Key Open Laboratory of Forest Genetics and Gene Engineering of National Forestry & Grassland, Nanjing Forestry University, Nanjing 210037, China

**Keywords:** *Pinus massoniana* Lamb., *PmNAC82*, secondary cell wall, transcription factor

## Abstract

The *NAC* transcription factor superfamily is one of the most prominent plant-specific regulatory gene families, extensively participating in multiple metabolic processes that govern plant growth, tissue development and stress adaptation. Masson pine (*Pinus massoniana* Lamb.) is a native dominant conifer widely cultivated across South China, whose timber resources possess great exploitation potential in pulp manufacturing and the paper industry. In this study, a total of 98 non-redundant NAC family members were mined at the genome-wide level. Functional validation revealed that *PmNAC82*, a member belonging to the VND evolutionary subgroup, acts as a core regulatory factor controlling wood formation. Subcellular localization tests confirmed PmNAC82 exclusively resides in the cell nucleus. Heterologous genetic transformation in poplar demonstrated that this gene positively regulates the accumulation of lignin and cellulose. Furthermore, through RT-qPCR, yeast one-hybrid assays, and EMSA, we confirmed that PmNAC82 can bind to the promoters of *PtrMYB3*, *PtrMYB21* and *PmCesA7*. These findings provide a solid foundation for further investigation into the molecular functions of *NAC* genes in Masson pine as well as their potential application towards molecular breeding strategies aimed at improving wood quality.

## 1. Introduction

Pine forest resources occupy an irreplaceable status in global terrestrial ecosystems, accounting for nearly 40% of the total forest area worldwide [1,2]. As an evergreen conifer naturally distributed in South China, Masson pine has long been regarded as one of the most vital commercial timber tree species in China. [3]. This conifer exhibits high economic value, and its softwood materials are widely applied in lumber processing, pulp production and papermaking industries [4]. Secondary cell walls (SCWs) constitute the main structural component of wood tissues. Hence, dissecting the transcriptional regulatory network underlying conifer secondary cell wall formation and wood development is of great theoretical significance for tree developmental biology research and practical value for forest biotechnology breeding.

Transcriptional regulatory switches in wood formation have been demonstrated for certain NAC proteins [5]. In *Arabidopsis*, a NAC subfamily capable of inducing SCW biosynthesis has been designated as the VNS family. Key regulators within this family, including *NST1* (secondary wall thickening promoting factor 1), *SND1* (secondary wall-associated NAC domain protein 1), *VND6* (vaso-associated NAC domain 6), and *VND7* (vaso-associated NAC domain 7), collaborate to orchestrate the entire SCW biosynthesis program [6,7], activating a cascade of downstream transcription factors involving MYB proteins. A similar transcriptional network involving WNDs (wood-associated NAC domain TFs) and MYBs has been described in Chinese fir, birch, moso bamboo, poplar, eucalyptus and pine [8,9,10,11,12,13,14,15,16,17,18]. In *Arabidopsis*, SND1 targets *MYB46* and *MYB83* are pivotal regulators of cellulose, hemicellulose, and lignin biosynthesis—three major components of secondary cell walls [9]. Several wood-associated MYBs, such as *EgMYB2* from *Eucalyptus grandis*, *PtrMYB3* and *PtrMYB20* from *Populus trichocarpa*, and *PtMYB1* and *PtMYB4* from *Pinus taeda*, are functional orthologs of *Arabidopsis MYB46* and *MYB83* [13,19,20,21,22]. Moreover, our previous study revealed that *PmMYB4* from Masson pine regulates SCW formation [23], suggesting its potential membership within a transcriptional cascade governing SCW formation in conifers.

Nevertheless, the lack of mature and stable genetic transformation technology severely restricts the progress of functional gene research in Masson pine. In the current work, PmNAC82 was identified as a putative ortholog of Arabidopsis VNS subfamily genes, with specific expression abundance in stem cambium. By constructing *PmNAC82* overexpression poplar lines, we verified that this gene can effectively accelerate lignin and cellulose deposition. Further molecular assays confirmed that three key functional genes related to secondary cell wall synthesis (*PtrMYB3*, *PtrMYB20* and *PtrCesA7*) are direct downstream targets of PmNAC82. Overall, this work provides novel research perspectives and technical references for the molecular biological study of Masson pine and establishes a molecular foundation for the genetic improvement of wood quality traits in this conifer tree.

## 2. Results

### 2.1. Genome-Wide Identification and Phylogenetic Profiling of NAC Family in Masson Pine

**Figure 1:**
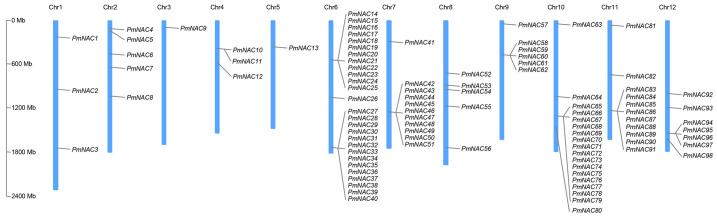
Chromosomal distribution pattern of the identified PmNAC family members in Masson pine.

**Figure 2:**
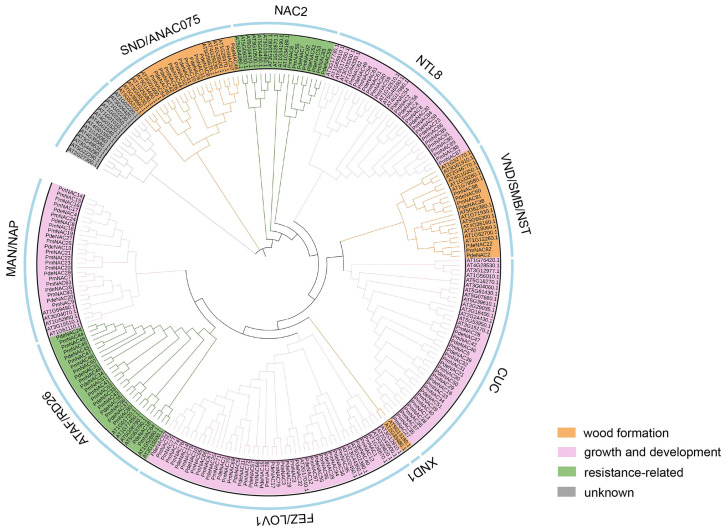
Evolutionary relationships of *NAC* transcription factors among *P*. *massoniana*, *A*. *thaliana* and *P*. *densiflora*. The phylogenetic tree contains 113 *Arabidopsis*, 62 *P. densiflora* and 98 *P. massoniana* NAC protein sequences. Functional grouping was conducted by referring to annotated *NAC* homologous genes in model plants.

Based on Pfam database annotation, 98 candidate *NAC* gene sequences were screened out and named *PmNAC1*-*PmNAC98* (Figure 1). The complete protein sequence information of all identified members is listed in Supplementary Table S1. A phylogenetic evolutionary tree was constructed using 113 NAC proteins from *Arabidopsis thaliana* and 62 NAC sequences from *Pinus densiflora*. According to evolutionary clustering results, all Masson pine *NAC* genes were classified into different subfamilies, and the potential physiological functions of each evolutionary branch were predicted (Figure 2).

### 2.2. Gene Structure, Conserved Domain and Motif Analysis of PmNAC Proteins

**Figure 3:**
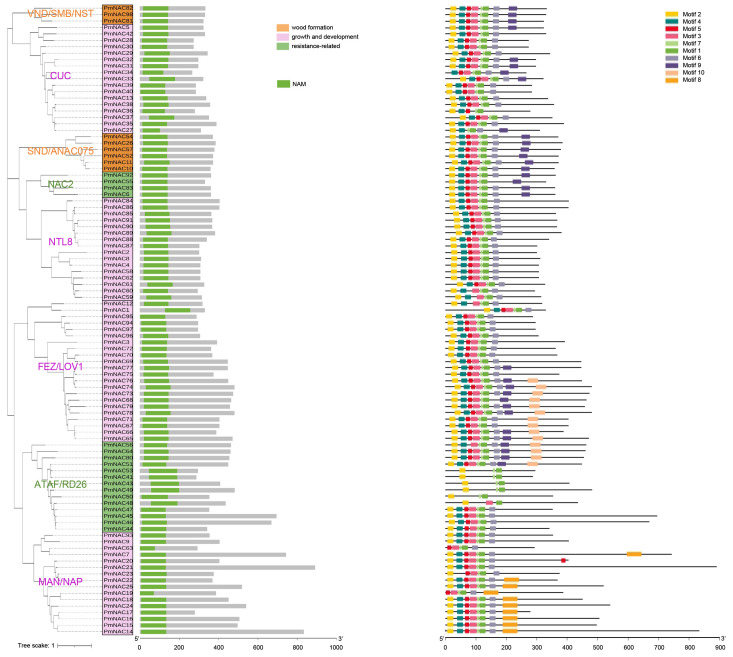
Conserved domain and motif analysis of PmNACs. All 98 PmNAC proteins harbored the conserved NAM domain, and ten conserved motifs (Motif 1 to Motif 10) were identified.

**Figure 4:**
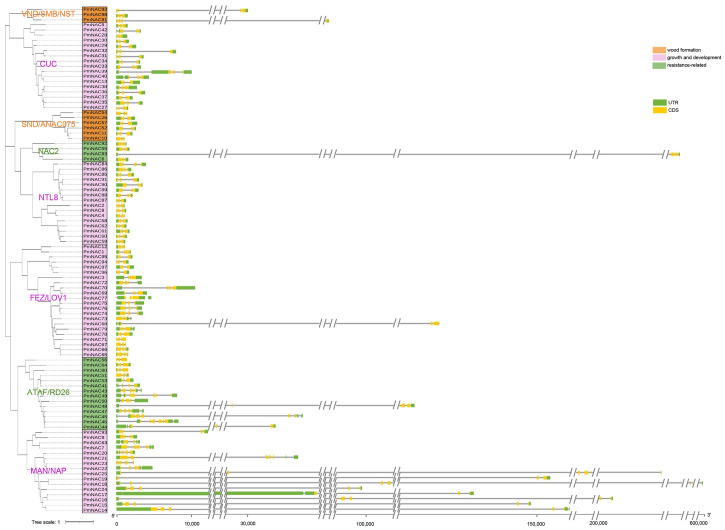
Structural characteristics of PmNAC family genes. Gray line segments represent intron regions.

To clarify the structural differentiation and evolutionary homology of *PmNAC* family genes, we systematically analyzed their conserved motif distribution and intron–exon composition based on phylogenetic clustering (Figure 3 and Figure 4). All 98 PmNAC proteins contain an intact NAM domain, which includes the classic NAC subdomain structures (NAC I-V). A total of 10 conserved functional motifs were predicted via the MEME online tool and designated Motif 1 to Motif 10 (Figure 3). Consistent with domain analysis outcomes, PmNAC members clustered on the same evolutionary branch present identical motif arrangement features, suggesting functional conservation among closely homologous genes.

Most PmNAC proteins exhibit a fixed arrangement of N-terminal motifs: Motif 2, 4, 5, 3, 7, 1 and 6, which perfectly matches the standard structural division of NAC subdomains (Figure S1). Intron–exon structure analysis revealed obvious structural divergence among PmNAC coding regions; some genes contain multiple untranslated regions, while others lack such non-coding segments entirely. This structural variation reflects the evolutionary differentiation process of the *PmNAC* gene family. Notably, members within the same phylogenetic clade share highly similar intron–exon organization, with only minor differences in the length of exon and intron sequences (Figure 4).

### 2.3. Genes from SND/ANA075 and VND/SMB/NST Clades Show Stem Cambium-Specific Expression

**Figure 5:**
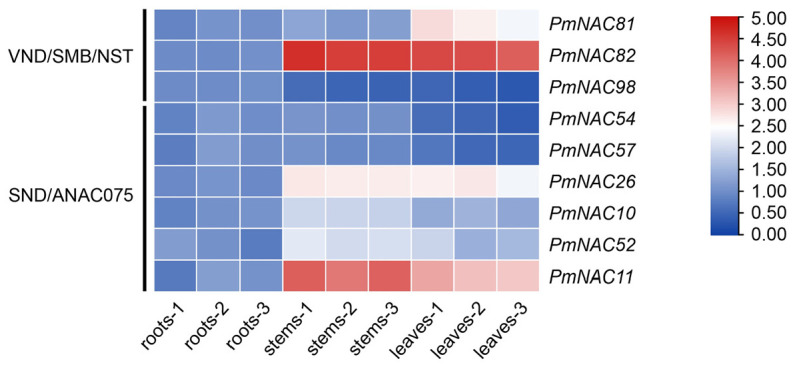
Tissue expression patterns of representative *PmNAC* genes. Relative expression levels were normalized to root tissue with *TUA* as internal reference gene. Data are presented as mean ± SE, with three independent biological replicates.

Phylogenetic tree analysis indicated nine PmNAC members clustered closely with VNS and SND subfamily branches (Figure 5). Real-time quantitative PCR was adopted to detect the tissue expression patterns of these candidate genes in 1-year-old Masson pine. Transcript profiling results showed that *PmNAC82* displayed extremely high expression specificity in stem cambium tissues, implying its core regulatory role in wood formation and warranting in-depth functional exploration (Figure 5).

### 2.4. PmNAC82 Protein Localizes Specifically to Cell Nucleus

**Figure 6:**
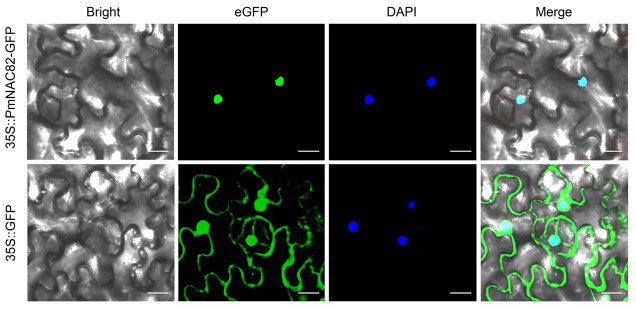
Subcellular localization of PmNAC82 in epidermal cells of *N*. *benthamiana*, bar = 20 μm.

Agrobacterium-mediated transient expression system in *Nicotiana benthamiana* leaves was used to determine the subcellular distribution of the PmNAC82 protein in vivo. Fluorescence microscopic observation showed that the green fluorescence signal of PmNAC82-eGFP fusion protein completely overlapped with the DAPI nuclear staining signal, proving that PmNAC82 is a nucleus-targeted transcription regulator (Figure 6).

### 2.5. PmNAC82 Overexpression Promotes Lignin and Cellulose Accumulation in Transgenic Poplar

**Figure 7:**
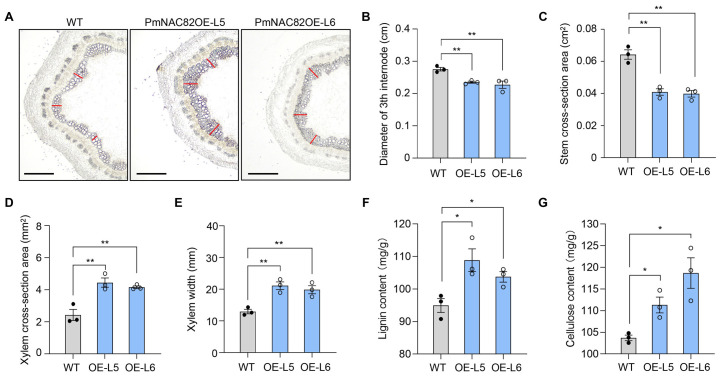
Overexpression of *PmNAC82* promotes the formation of SCW in poplar. (**A**) Stem cross-section under optical microscopy; the full stem diameter is not shown. The red line indicates the position where xylem thickness was measured. Scale bar = 500 μm. Measurement of the 3rd internode diameter (**B**), stem cross-section area (**C**), xylem cross-section area (**D**), xylem width (**E**), lignin content (**F**), and cellulose content (**G**). Each set of data was obtained from three biological replicates with three technical replicates. Black solid circles and open circles represent individual biological replicate data points of the wild type (WT) and overexpression lines (OE-L5/OE-L6), respectively. Asterisks represent significant differences compared with the wild type based on one-way ANOVA (* *p* < 0.05, ** *p* < 0.01).

RT-PCR and qRT-PCR detection verified the successful integration and stable expression of *PmNAC82* in ten independent transgenic poplar lines (Figure S2). Two lines (L5 and L6) with the highest transcript abundance were selected for subsequent phenotypic observation and physiological index determination (Figure S3). Two months after soil transplantation, significant differences in plant height and xylem anatomical structure were observed between transgenic lines and wild-type poplar (Figure S4). Transgenic plants showed reduced stem thickness (Figure 7A–C), while xylem thickness and cross-sectional area ratio increased markedly (Figure 7D,E). Physiological measurement further confirmed that heterologous expression of *PmNAC82* significantly enhanced lignin and cellulose accumulation in poplar (Figure 7F,G).

**Figure 8:**
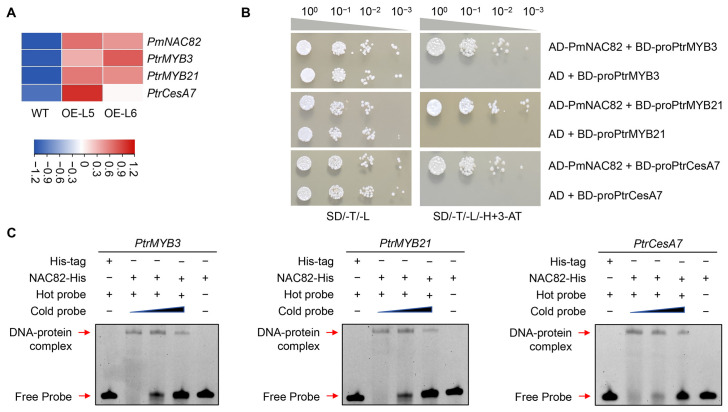
PmNAC82 directly binds and regulates *PtrMYB3*, *PtrMYB21* and *PtrCesA7*. (**A**) Transcript levels of target genes in PmNAC82 transgenic lines and wild type. Relative expression was normalized to leaf tissue with *PdbActin* (Potri.001G309500) as internal reference. Data represent mean ± SE with three biological replicates. (**B**) Y1H assay verifying protein–promoter interaction, yeast cultured on SD/-Trp/-Leu and SD/-Trp/-Leu/-His selective media. (**C**) EMSA detection of PmNAC82 binding to SNBE motifs in gene promoters. Biotin-labeled SNBE probes were incubated with empty HIS-tag protein or PmNAC82-HIS fusion protein. Mobility shift in wild-type probes rather than mutant ones confirmed specific binding activity.

To elucidate the molecular mechanism by which *PmNAC82* regulates wood formation and component deposition, we detected the transcription levels of secondary wall synthetic-related genes via qRT-PCR. The results indicated that key functional genes including *PtrMYB3*, *PtrMYB21* and *PtrCesA7* were significantly upregulated in transgenic lines (Figure 8A). It is suggested that ectopic expression of *PmNAC82* can extensively reshape the transcriptional regulatory pattern of secondary cell wall-related genes in poplar.

### 2.6. PmNAC82 Activates the Expression of PtrMYB3, PtrMYB21, and PtrCesA7

RT-qPCR detection showed that heterologous overexpression of *PmNAC82* in poplar remarkably elevated the transcription abundance of *MYB3*, *MYB21* and *CesA7* (Figure 8A). Yeast one-hybrid assay was further carried out to verify the binding interaction between PmNAC82 and the promoter regions of these three genes. After adding 3-AT to selective medium, control yeast strains showed complete growth inhibition, while co-transformed experimental strains grew normally, demonstrating that PmNAC82 can bind to the promoter sequences of target genes in yeast cells (Figure 8B). Meanwhile, full-length PmNAC82 protein fused with His-tag was purified for EMSA detection (Figure S5). EMSA results further confirmed that the recombinant PmNAC82 protein could specifically bind to the promoter fragments of *PtrMYB3*, *PtrMYB21* and *PtrCesA7* (Figure 8C). In summary, these experimental data prove that PmNAC82 directly targets the promoters of the three downstream genes and initiates their transcriptional expression.

## 3. Discussion

The *NAC* transcription factor family constitutes one of the largest and most evolutionarily conserved families of plant-specific transcription regulators, with members distributed across all land plant lineages from bryophytes to angiosperms. Members of this family participate in a wide range of fundamental biological processes, including embryogenesis, organ development, leaf senescence, hormone signaling, abiotic stress responses, xylem cell differentiation, and secondary cell wall biosynthesis. Among these functions, the regulatory roles of NAC proteins in vascular development and cell wall formation are particularly critical for woody plants, as they directly determine wood structure, chemical composition, and biomass productivity—traits that are of great economic and ecological significance for forestry production [24,25,26,27]. Despite their essential roles in plant growth and development, genome-wide or transcriptome-wide identification and functional analysis of *NAC* family genes remain relatively limited in most conifer species, especially in non-model gymnosperms such as Masson pine, which is an economically important evergreen conifer widely cultivated in southern China for timber and resin production.

In this study, we systematically identified a total of 98 distinct *PmNAC* genes from the transcriptome of Masson pine using bioinformatics methods, including sequence alignment, conserved domain analysis, and phylogenetic reconstruction (Figure 2, Figure 3 and Figure 4; Table S1). This number is significantly higher than those reported in other conifers, such as maritime pine (*P. pinaster*, 37 *NAC* genes) [28] and red pine (*P. densiflora*, 62 *NAC* genes) [14]. The higher number of *PmNAC* genes identified in our study may be attributed to the comprehensiveness of the transcriptome data used, which covered multiple tissues and developmental stages of Masson pine, ensuring the capture of a broader range of *NAC* gene transcripts. In a large-scale comparative analysis covering 160 plant species, Mohanta et al. (2020) reported average *NAC* gene numbers of 141.20 in monocots, 125.04 in eudicots, 75 in gymnosperms, and only 22.66 in bryophytes, suggesting a gradual expansion of the NAC family during plant evolution, which may be associated with the increasing complexity of plant body plans and adaptation to diverse environments [29,30,31]. The number of *PmNAC* genes identified in this study is close to the average level of gymnosperms and provides a relatively complete gene resource, indicating that our identification is comprehensive and reliable for further evolutionary and functional investigations, laying a solid foundation for exploring the role of *NAC* genes in Masson pine wood formation.

Accumulating evidence has demonstrated that the VNS subfamily, which includes the VND (Vascular-Related NAC-Domain), NST/SND (NAC Secondary Wall Thickening Promoting Factor/SND1), and SMB (Secondary Wall-Associated NAC Domain Protein) clades, functions as the master regulatory switches initiating the transcriptional cascade responsible for xylem cell specification and SCW deposition in both herbaceous and woody plants. These top-level regulators are widely considered to be the core initiators of the entire SCW biosynthetic pathway, as they can directly activate the expression of downstream transcription factors and structural genes involved in cellulose, hemicellulose, and lignin biosynthesis. In recent years, several VNS orthologs have been functionally characterized in coniferous species, providing valuable insights into the conservation and divergence of SCW regulatory mechanisms between gymnosperms and angiosperms.

Akiyoshi et al. (2020) identified five *PtaVNS* genes (*PtaVNS1-5*) from loblolly pine and verified that *PtaVNS1*, *2*, *3*, and *5* act as transcriptional regulators capable of inducing tracheary element differentiation through transient overexpression in tobacco leaves and dual-luciferase reporter assays, indicating their conserved role in xylem development [32]. Similarly, Kim et al. (2021) reported that *PdeNAC2* is specifically expressed in the secondary xylem of *P. densiflora*. Its ectopic expression in tobacco and *Arabidopsis* leaves can induce the formation of xylem vessel-like cells [14]. Such ectopic overexpression further confirms the functional conservation of *VNS* genes among different plant lineages. In maritime pine, *PpNAC1*, an ortholog of *Arabidopsis SND1*, was identified as a key regulator of the phenylpropanoid pathway and lignin biosynthesis, highlighting the specific role of *VNS* genes in conifer SCW formation [15]. In the present study, phylogenetic analysis revealed that *PmNAC81*, *PmNAC82*, and *PmNAC98* were grouped into the VND/SMB/NST subfamily, whereas *PmNAC10*, *11*, *26*, *52*, *54*, and *PmNAC57* belonged to the SND/ANAC075 clade (Figure 2). These distribution patterns strongly suggest that these *PmNAC* genes may serve as core regulators involved in SCW biosynthesis and wood formation in Masson pine. RT-qPCR analysis further revealed that *PmNAC82* was predominantly expressed in the stem cambium region (Figure 5), which is the primary site of xylem cell differentiation and SCW deposition. This expression pattern is highly consistent with the expression patterns of *PtrWNDs* (Wood-Associated NAC Domain) in poplar and *SND1*, *NST1*, *VNDs*, and *SMB* in *Arabidopsis* [33,34], further supporting its potential role in regulating vascular development and secondary wall thickening.

In *P. trichocarpa*, overexpression of wood-associated NAC domain (*WND*) genes significantly promotes SCW thickening in stem tissues but simultaneously restricts cell elongation and overall plant growth, which is a common phenotypic effect of enhancing SCW biosynthesis in woody plants [35]. To characterize the biological function of *PmNAC82*, we generated *PmNAC82*-overexpressing transgenic poplar plants, using poplar as a heterologous expression system due to its short generation time and well-characterized SCW regulatory network. Phenotypic and histological observations showed that overexpression of *PmNAC82* significantly promotes xylem thickening (Figure 7A–E). Correspondingly, the transcription levels of multiple structural genes involved in cellulose, hemicellulose, and lignin biosynthesis were significantly elevated in transgenic lines (Figure 8A), including genes encoding cellulose synthases (*CesA*), *MYB3*, and *MYB21*, which are key enzymes in SCW component biosynthesis.

Previous studies have confirmed that these structural genes are indispensable for lignin polymerization, cellulose crystallization, and cell wall component assembly; their repression or mutation directly leads to altered lignin content, monomer composition, and cell wall thickness, which can affect wood quality and mechanical properties [36,37,38]. Importantly, we also observed significantly increased expression of *PtrMYB3* and *PtrMYB21*, which are orthologs of *PmMYB4* and *Arabidopsis MYB46/MYB83* (Figure 8A). These *MYB* transcription factors are specifically expressed in secondary xylem and function as key secondary-level regulators in the NAC-mediated SCW regulatory cascade, acting as a bridge between NAC master switches and structural genes [20,39]. Furthermore, Y1H and EMSA analyses revealed that PmNAC82 directly binds to the promoters of *PtrMYB3* and *PtrMYB20* (Figure 8B,C). Taken together, these results indicate that the VNS-MYB-dependent transcriptional regulatory network controlling SCW formation is highly conserved between poplar and Masson pine, reflecting the evolutionary conservation of this regulatory pathway across woody plant lineages.

**Figure 9:**
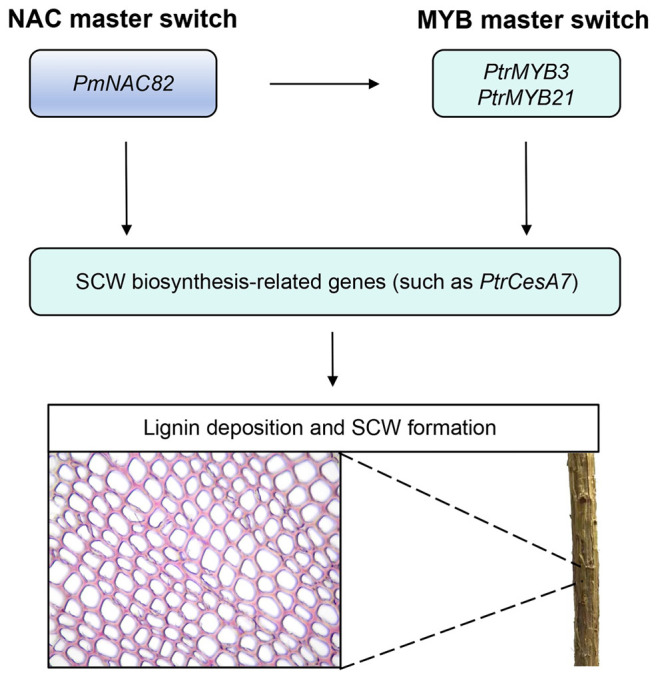
Molecular model of *PmNAC82* regulating secondary cell wall biosynthesis.

Based on all the above findings, we propose a functional model illustrating the molecular role of *PmNAC82* during SCW formation in Masson pine. *PmNAC82* acts as a core upstream transcription factor that directly binds to the SNBE (Secondary Wall NAC Binding Element) motifs in the promoters of downstream target genes, including *PtrMYB3*, *PtrMYB20*, and *PtrCesA7*, thereby activating their expression (Figure 9). This activation further enhances the biosynthesis of lignin and cellulose, the major components of SCW, and promotes SCW deposition in xylem cells. These findings not only confirm the evolutionary conservation of the primary NAC master switch system governing wood formation but also deepen our understanding of the complex and precise transcriptional regulatory network in conifers, which has been less studied compared to angiosperms.

Uncovering the function of *PmNAC82* provides novel insights into the regulatory mechanism of wood formation in gymnosperms and offers a promising target for the genetic improvement of wood yield and quality in Masson pine through molecular breeding and biotechnology strategies. Additionally, this study provides a valuable gene resource for further exploring the functional diversity of *NAC* genes in conifers, and future studies could focus on the functional characterization of other *PmNAC* genes identified in this study, as well as the interaction between *PmNAC82* and other transcription factors or structural genes in the SCW regulatory network.

## 4. Materials and Methods

### 4.1. Identification of NAC Genes in the P. massoniana Genome

Referring to the NAM conserved domain (PF02365), the hidden Markov model file was downloaded from the Pfam database. HMMER 3.3.2 software (Chevy Chase, MD, USA) was used to mine NAC protein sequences from Masson pine Iso-Seq data [40], with the E-value threshold set as E < 10^−3^ under default operation parameters. Pfam 37.0 (Hinxton, UK) and CD-search v3.20 online tools (Bethesda, MD, USA) were jointly used to screen sequences containing complete NAM domain. Finally, redundant sequences with similarity over 97% were eliminated, and 98 non-redundant PmNAC protein sequences were obtained.

### 4.2. Amino Acid Feature Analysis, Multiple Sequence Alignment and Phylogenetic Construction

The molecular weight and isoelectric point of each PmNAC protein were predicted via ExPASy 3.0 online platform (Geneva, Switzerland). Conserved domain characteristics were analyzed using ExPASy-Prosite and visualized by TBtools-II (version 2.303) software (Guangzhou, China). The MEME program was applied to identify conserved functional motifs, and results were displayed with TBtools. Multiple protein sequence alignment was performed by DNAMAN 10.0 (Saint-Ferréol-les-Neiges, QC, Canada) and ClustalX2.1 (Dublin, Ireland) with default parameters. Based on NAM domain amino acid sequences from *A. thaliana*, *P. densiflora* and *P. massoniana*, a maximum likelihood phylogenetic tree was constructed using MEGA-X 10.2.6 (Philadelphia, PA, USA), with 1000 bootstrap replicates for reliability evaluation [41].

### 4.3. Plant Materials and Cultivation Conditions

Plant materials included 1-year-old and 1-month-old Masson pine seedlings which were preserved in Nanjing Forestry University. Tobacco and poplar seedlings were cultivated in an artificial climate incubator at a constant 22 °C, under a 16 h light/8 h dark photoperiod. Tobacco seeds were surface sterilized with 10% NaClO solution for 15 min and sown on 1/2 MS solid medium. After two days of dark vernalization at 4 °C, materials were transferred to normal culture condition. Nine days after sowing, seedlings were transplanted into a mixed substrate composed of 60% peat soil, 30% perlite and 10% vermiculite.

### 4.4. RNA Extraction and Real-Time Quantitative PCR Detection

Total RNA was isolated using FastPure Universal Plant Total RNA Isolation Kit (Vazyme Biotech, Nanjing, China) [42]. RNA concentration and purity were determined by NanoDrop 2000 (Thermo Fisher Scientific, Waltham, MA, USA), and integrity was detected via 1.2% agarose gel electrophoresis. First-strand cDNA was synthesized using ClonExpress II One Step Cloning Kit (Vazyme, Nanjing, China) [43]. qPCR specific primers were designed by Primer 5.0 (Table S2). The 10 μL reaction system contained 1 μL 20-fold diluted cDNA, 5 μL SYBR Green Master Mix, 0.4 μL forward and reverse primer (10 μM each), and 3.2 μL sterile ddH_2_O. PCR amplification program: 95 °C pre-denaturation for 60 s; 40 cycles of 95 °C 15 s, 60 °C 15 s, and 72 °C 10 s; followed by melting curve analysis from 60 °C to 95 °C. The TUA gene was used as internal reference [44]. Each sample was set with three biological replicates and three technical replicates. Relative gene expression was calculated by the 2^−∆∆Ct^ method [45], and statistical significance was analyzed by *t*-test and one-way ANOVA with SPSS 26.0 software (Armonk, NY, USA) (* *p* < 0.05, * *p* < 0.01).

### 4.5. Subcellular Localization Assay

The full-length CDS sequence of *PmNAC82* was inserted into a pCAMBIA1305-eGFP expression vector (Table S3) to construct 35S::PmNAC82-eGFP fusion vector. The recombinant plasmid was transformed into *N. benthamiana* leaves via the Agrobacterium-mediated infiltration method [46]. Fluorescence distribution was observed and photographed using a Zeiss LSM 710 confocal laser scanning microscope (Zeiss, Jena, Germany).

### 4.6. Agrobacterium-Mediated Poplar Genetic Transformation

The full-length ORF of *PmNAC82* was inserted into the pBI121 vector downstream of 35S promoter using *XbaI* and *SmaI* restriction sites. The constructed recombinant plasmid was transformed into *Agrobacterium tumefaciens* strain GV3101 and then introduced into wild-type poplar via the leaf disk transformation method [47].

### 4.7. Plant Growth Index Measurement

Plant height (from stem base to apical growing point) and the 3rd internode diameter were measured in three-month-old transgenic and wild-type poplar plants. Three independent lines were selected, with three technical replicates for each line.

### 4.8. Microscopy and Histochemistry

The 3rd internode stems of three-month-old poplar were collected to prepare paraffin sections following standard experimental protocols [48]. ImageJ 1.54f software (Bethesda, MD, USA) was used to quantify xylem cell morphological parameters and cell wall thickness. For histological analysis, stem samples were fixed in 4% paraformaldehyde solution (prepared with 0.1 M phosphate buffer, pH 7.4) under vacuum treatment for 15 min, repeated three times. After dehydration and paraffin infiltration for 5 days, 10 μm continuous thin sections were prepared using a microtome.

### 4.9. Determination of Lignin and Cellulose Content

Total lignin content was measured by the acetyl bromide method [49]. Cellulose content was detected following the Van Soest determination protocol [49]. The average content of two components was calculated based on three technical replicate data.

### 4.10. Yeast One-Hybrid Assay

The CDS of PmNAC82 was inserted into the AD vector, while promoter fragments of *PtrMYB3*, *PtrMYB20* and *PtrCesA7* were ligated into the pHIS2 vector. Primers for vector construction are listed in Table S3. The recombinant pHIS2 plasmid and empty AD vector were co-transformed into yeast strain AH109 and cultured on SD/-Leu-Trp-His medium supplemented with gradient concentration of 3-AT to inhibit background self-activation [19]. The interaction between PmNAC82 and target gene promoters was judged by yeast growth status on selective medium.

### 4.11. Electrophoretic Mobility Shift Assay (EMSA)

The EMSA experiment was carried out in accordance with standard laboratory procedures [50]. The *PmNAC82* CDS was fused into the pET-28a prokaryotic expression vector, and fusion protein was induced and purified in *E. coli* BL-21 Gold competent cells for subsequent EMSA detection. Promoter fragments containing complete SNBE motifs of target genes were synthesized and labeled with biotin at 5′ end (Table S3); unlabeled identical fragments were used for competitive binding control. The EMSA reaction was performed using Beyotime Chemiluminescent EMSA Kit (Beyotime Biotechnology (Shanghai, China) according to the manufacturer’s instructions. Finally, DNA probes were transferred to the nitrocellulose membrane and detected by the chemiluminescence imaging system.

### 4.12. Statistical Analysis

All experimental data were processed using GraphPad Prism v8.0.2 software. All tests were set with at least three independent biological replicates. Student’s *t*-test was used for two-group difference analysis, and one-way ANOVA was adopted for multi-group significance comparison (* *p* < 0.05, ** *p* < 0.01).

## 5. Conclusions

In this study, 98 non-redundant *PmNAC* genes (named *PmNAC1*-*PmNAC98*) were systematically identified from Masson pine transcriptome data via bioinformatic approaches and classified into multiple subfamilies through phylogenetic analysis combined with NAC sequences of *A. thaliana* and *P. densiflora*. Structural analysis showed that homologous PmNAC members share highly conserved motif composition and intron–exon arrangement patterns, reflecting functional conservation within the same evolutionary clade. Among nine PmNAC members clustered with secondary cell wall-related VNS/SND subfamilies, *PmNAC82* belongs to the VND subgroup, localizes to the cell nucleus, and exhibits specific high expression in stem cambium tissues. Transgenic poplar verification confirmed that *PmNAC82* facilitates xylem formation by directly binding to the promoters of key synthetic genes (*PtrMYB3*, *PtrMYB21*, *PtrCesA7*) and activating their transcriptional expression. This research enriches the *NAC* gene resource database of coniferous plants and provides an important candidate gene for the molecular genetic improvement of wood quality traits in Masson pine.

## Data Availability

The original contributions presented in this study are included in the article/Supplementary Materials. Further inquiries can be directed to the corresponding authors.

## Supplementary Materials

The following supporting information can be downloaded at: https://www.mdpi.com/article/10.3390/plants15101568/s1, Figure S1: Details of conserved motifs from PmNAC proteins; Figure S2: Genomic PCR confirmation of the *PmNAC82* gene in transgenic poplar lines; Figure S3: Expression of *PmNAC82* in transgenic lines and WT. Relative gene expression was measured using qPCR with *PtrActin* (Potri.001G309500) as a reference gene, prior to normalization to the expression levels in leaves. Data are shown as mean ± SE, with three biological replicates in the experiment. ** *p* < 0.01, * *p* < 0.05, Student’s *t*-test; Figure S4: Phenotype of poplar overexpressing *PmNAC82*. Bar = 5 cm; Figure S5: Expression and purification of the recombinant PmNAC82-His protein in *E. coli*.; Table S1: The protein sequences of 98 PmNACs proteins; Table S2: Sequences of primer pairs used for qRT-PCR analysis; Table S3: Sequences of primer pairs used for vector construction and transgenic plants sequencing.
