# Supplementary material for: Identification of the PmNAC Gene Family in Pinus massoniana: PmNAC82 Modulates Wood Biosynthesis by Activating SCW-Related Genes
Source: Plants (Basel). 2026 May 21;15(10):1568. doi: 10.3390/plants15101568 (PMC13210743; doi:10.3390/plants15101568)
Supplement: Supplementary file 1 [file plants-15-01568-s001.zip › plants-4295536-supplementary.pdf]

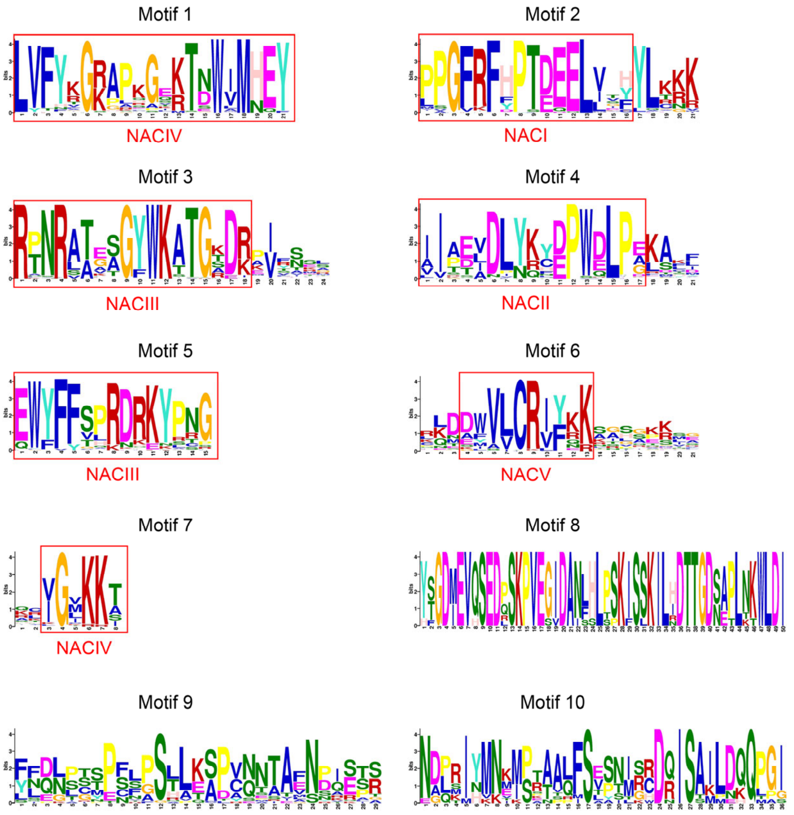

Figure S1. Details of conserved motifs from PmNAC proteins.

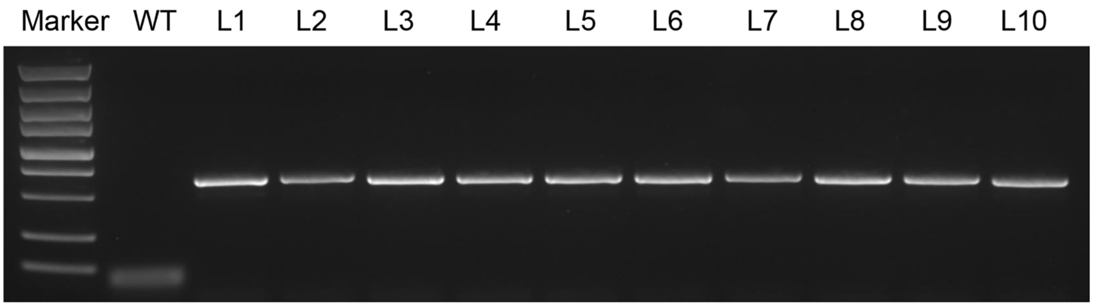

Figure S2. Genomic PCR confirmation of the *PmNAC82* gene in transgenic poplar lines.

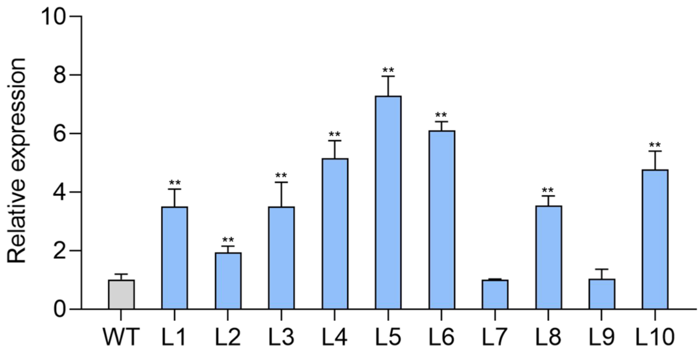

Figure S3. Expression of *PmNAC82* in transgenic lines and WT. Relative gene expression was measured using qPCR with *PtrActin* (Potri.001G309500) as a reference gene, prior to normalization to the expression levels in leaves. Data are shown as mean ± SE, with three biological replicates in the experiment. ** $p < 0.01$, * $p < 0.05$, Student's *t*-test.

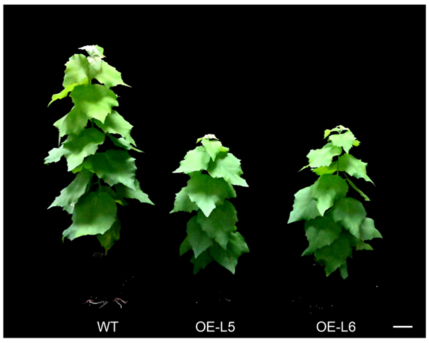

Figure S4. Phenotype of poplar overexpressing *PmNAC82*. Bar = 5 cm.

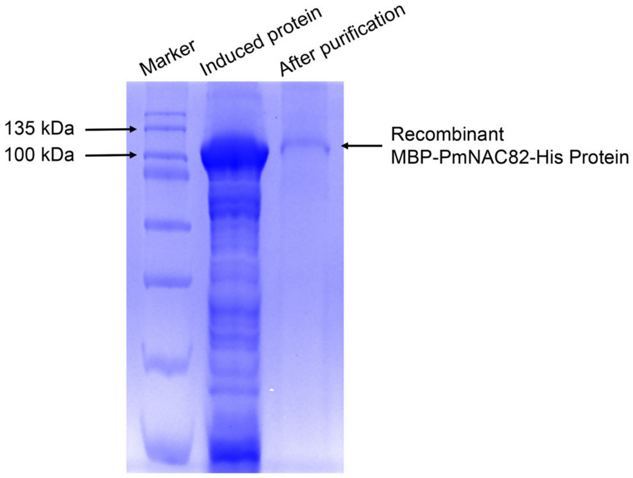

Figure S5. Expression and purification of the recombinant PmNAC82-His protein in *E. coli*.
